# H11/HspB8 and Its Herpes Simplex Virus Type 2 Homologue ICP10PK Share Functions That Regulate Cell Life/Death Decisions and Human Disease

**DOI:** 10.1155/2012/395329

**Published:** 2012-09-27

**Authors:** Laure Aurelian, Jennifer M. Laing, Ki Seok Lee

**Affiliations:** Department of Pharmacology, University of Maryland School of Medicine, 655 West Baltimore Street, Baltimore, MD 21201-1559, USA

## Abstract

Small heat shock proteins (sHsp) also known as HspB are a large family of widely expressed proteins that contain a 90 residues domain known as **α**-crystallin. Here, we focus on the family member H11/HspB8 and its herpes simplex virus type 2 (HSV-2) homologue ICP10PK, and discuss the possible impact of this relationship on human disease. H11/HspB8 and ICP10PK are atypical protein kinases. They share multi-functional activity that encompasses signaling, unfolded protein response (UPR) and the regulation of life cycle potential. In melanocytes H11/HspB8 causes growth arrest. It is silenced in a high proportion of melanoma prostate cancer, Ewing's sarcoma and hematologic malignancies through aberrant DNA methylation. Its restored expression induces cell death and inhibits tumor growth in xenograft models, identifying H11/HspB8 as a tumor suppressor. This function involves the activation of multiple and distinct death pathways, all of which initiate with H11/HspB8-mediated phosphorylation of transforming growth factor **β**-activated kinase 1 (TAK1). Both ICP10PK and H11/HspB8 were implicated in inflammatory processes that involve dendritic cells activation through Toll-like receptor-dependent pathways and may contribute to the onset of autoimmunity. The potential evolutionary relationship of H11/HspB8 to ICP10PK, its impact on human disorders and the development of therapeutic strategies are discussed.

## 1. Introduction

Small Hsp (sHsp) also known as HspB are a large family of widely expressed proteins that contain a 90-residue domain known as *α*-crystallin [1]. The 10 members of the human HspB family (17.0–28.4 kDa in size) are expressed widely and function as molecular chaperones, which restore protein folding and cellular homeostasis [2]. They have a flexible quaternary structure that allows structural changes, which alter their oligomeric substructure and interaction with target proteins depending on intracellular conditions and posttranslational modifications [3]. Current understanding of the HspB structure and function has recently been reviewed [4, 5]. Here, we focus on the family member H11/HspB8 and its herpes simplex virus type 2 (HSV-2) homologue known as ICP10PK and discuss the possible impact of this relationship on human disease.

## 2. HSV-2 Encodes Hsp Homologues with Antiapoptotic Activity

To the extent of our knowledge, HSV-2 is the only human viral pathogen that encodes Hsp homologues. One of these is UL14 that has 27% sequence identity with the protein-binding domain of Hsp70, but lacks ATPase activity. Like Hsp70, UL14 undergoes nuclear translocation after heat shock, it appears to aid in protein folding, and it has antiapoptotic activity [6]. The second HSV-2 protein that is an Hsp homologue is ICP10PK. It has 32% sequence identity with H11/HspB8, in the catalytic core and they share a number of unique functional properties. 

### 2.1. ICP10PK Has Intrinsic Kinase Activity

**Figure 1:**
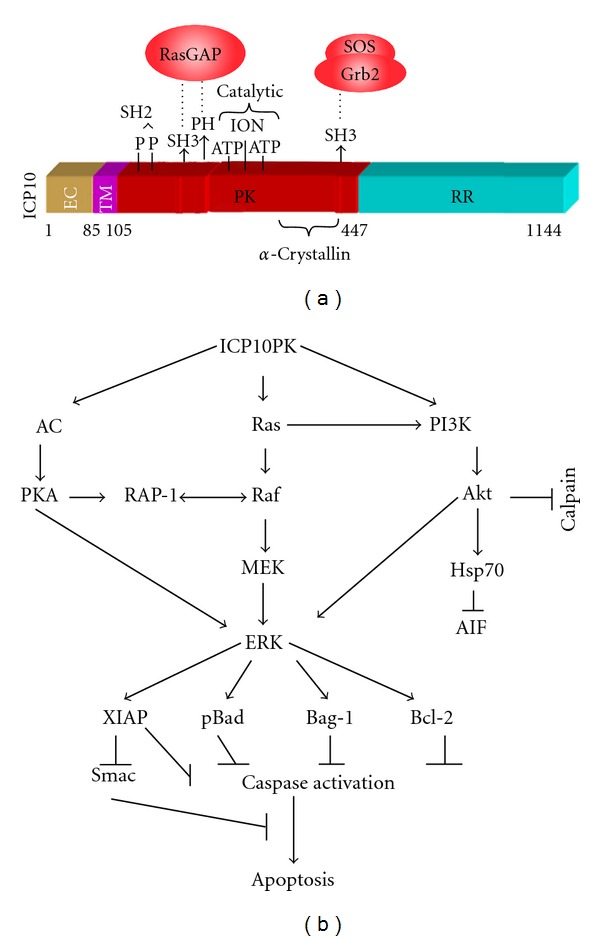
ICP10PK activates survival/proliferative pathways that initiate with Ras activation. (a) Schematic representation of the ICP10PK functional motifs. Shown are the binding sites for the Grb2-Sos complex and Ras-GAP, the PK catalytic motifs the TM and extracellular (EC) domains, and the *α*-crystallin domain. (b) The activated signaling pathways include the Ras/c-Raf/MEK/ERK, the PI3-K/Akt, and the AC/PKA. The pathways converge on ERK activation and modulate apoptosis regulatory proteins that control caspase activation (Bcl2 family) and caspase activity (iAP family) and their inhibitors (Smac). ICP10PK also inhibits calpain activation and AIF release. Ras activation is through recruitment of the GEF Sos and inhibition of the Ras-GAP through direct phosphorylation [10–13].

ICP10PK is a serine/threonine kinase that is fused in frame to the large subunit of the HSV-2 ribonucleotide reductase (R1, also known as ICP10) [7]. Phylogenetic analyses indicate that ICP10PK is a member of a family of atypical PKs (aPKs), also known as pseudokinases [8]. These kinases retain the 3 motifs required for kinase activity but lack one or more residues retained by the classical eukaryotic PKs. They can also function as scaffold proteins or allosteric activators [9, 10]. Pseudokinases do not always display catalytic activity but they are widely located in every branch in the phylogenetic tree, suggesting that they function like the eukaryotic PKs [10]. The ICP10PK functional domains are schematically represented in Figure 1(a). The catalytic core contains the 3 motifs essential for enzymatic activity that are best conserved relative to the eukaryotic PKs, including the Gly-rich loop (catalytic motif I), which is involved in stabilizing nontransferable ATP *β*-phosphates, and the two conserved charged residues that, respectively, constitute catalytic motifs II (Lys) and III (Glu) [11, 12]. The core is preceded by a transmembrane (TM) helical segment that is required for kinase activity, likely related to the conformational context that results from its anchorage into the plasma membrane [11–13]. It is followed by a degenerate *α*-crystallin motif [14] that establishes ICP10PK as being evolutionarily related to the HspB family [1].

Significantly, despite its overall similarity to HSV-2, HSV-1 has an R1 protein that contains a poorly conserved additional N-terminal domain, which lacks kinase activity [7, 8]. While the failure to detect kinase activity in the HSV-1 R1 protein has been interpreted to indicate that the kinase activity of ICP10 is due to contamination by casein kinase [15], available data provide incontrovertible evidence that the ICP10 kinase activity is intrinsic. Specifically, (i) kinase activity was lost through replacement of the Lys and/or Glu residues in catalytic motifs II and III or deletion of the TM domain, (ii) ICP10 binds the ^14^C-labeled ATP analogue FSBA, and binding is specifically competed by another ATP analogue, AMP-PNP, (iii) treatment with epidermal growth factor activates the kinase activity of a chimeric protein consisting of the ligand-binding domain of the epidermal growth factor receptor and the PK domain of ICP10, and (iv) the ICP10 kinase activity favors Mn^2+^ ions, does not require monovalent cations, and is not inhibited by zinc sulfate, all of which are properties distinct from those of the putative contaminant [11, 12]. In fact, the HSV-1 R1 protein (ICP6) lacks kinase activity under conditions that allow for the putative contamination impugned for ICP10 [7]. 

### 2.2. ICP10PK Is Required for HSV-2 Growth

ICP10PK is required for HSV-2 growth and latency reactivation [16–19]. This is related to its ability to inhibit neuronal apoptosis caused by proapoptotic viral genes and it involves the activation of survival pathways, notably Ras/MEK/ERK [20–27] (see Section 3 for further details). By contrast, both HSV-1 and a HSV-2 mutant deleted in ICP10PK (ΔPK) cause neuronal apoptosis [21] and activate the stress JNK/c-Jun pathway, which is required for their optimal replication ([28], Colunga and Aurelian, unpublished). The activated JNK/c-Jun pathway and neuronal apoptosis are involved in HSV-1-mediated adult encephalitis [29], which is the most common viral encephalitis. Encephalitis in adults is virtually never caused by HSV-2, likely related to the ability of ICP10PK to override the JNK/c-Jun-induced apoptosis [21]. The findings that (i) HSV-1 lacks Hsp homologues, (ii) the two viruses have distinct amino-terminal R1 domains, and (iii) HSV-1 activates stress as opposed to survival pathways in order to support its replicative cycle are indicative of a divergent evolution for these two largely similar viruses. 

## 3. ICP10PK Activates Survival Pathways That Inhibit Apoptosis

ICP10PK behaves as a constitutively activated growth factor receptor that activates multiple survival/proliferative pathways (Figure 1(b)). Ras activation is a pivotal function. ICP10PK activates Ras both by recruiting the guanine nucleotide exchange factor (GEF) Sos and inactivating the Ras inhibitor Ras-GAP. To recruit Sos to the membrane and bring it into the proximity of Ras, ICP10PK binds the SRC homology 3 (SH3) domain of the adaptor protein Grb2 in the Grb2-Sos complex. To inactivate Ras-GAP, ICP10PK binds and phosphorylates it. Once it is activated, Ras further activates the downstream Raf/MEK/ERK and PI3-K/Akt pathways. In addition to these two major survival pathways, ICP10PK also upregulates/stabilizes adenylate cyclase (AC) and activates PKA, upregulates the Ras family member Rap-1, activates the B-Raf kinase activity, inhibits calpain activation and blocks the release of apoptosis-inducing factor (AIF), and upregulates/stabilizes Hsp70. Downstream signaling includes (i) increased activation/stability of the transcription factor CREB, (ii) stabilization of the antiapoptotic protein Bcl-2 and the co-chaperone Bag-1, (iii) inactivation of the proapoptotic protein Bad through phosphorylation, and (iv) MEK-dependent upregulation of the antiapoptotic protein XIAP and downregulation of the apoptogenic protein Smac/DIABLO. In normal diploid cells, these ICP10PK functions are mitogenic (induce cell proliferation). In immortalized cells, they cause neoplastic transformation. However, in postmitotic neurons they inhibit programmed cell death (PCD) caused by different stress conditions, including HSV-1 infection [21–27]. Because cancer cells have constitutively activated Ras and PI3-K pathways, the ICP10PK deleted mutant ΔPK has potent oncolytic activity, which is based on cancer cell lysis through selective virus replication. Significantly, the ΔPK oncolytic activity also includes the activation of multiple programmed cell death (PCD) pathways and is accompanied by the upregulation of H11/HspB8 that functions as a tumor suppressor [30]. The role of the *α*-crystallin motif in these ICP10PK functions is still unknown. 

## 4. H11/HspB8 Cloning and DNA Methylation

H11/HspB8 was cloned in our laboratory using ICP10PK as a probe and called H11 [31, 32]. It is also known as Hsp22 [33] and the product of the *E2IG1 *gene expressed in estrogen-treated breast cancer cells [34]. H11/HspB8 has 32% identity and 59% homology to ICP10PK in the catalytic core [14, 31]. It was classified as an sHsp based on the presence of an *α*-crystallin sequence, which is believed to provide *sine qua non *evidence of evolutionary relationship, even when characteristic residues in the sequence have been lost [1]. Using FISH and M-FISH we originally reported that H11/HspB8 maps at position 12q24.1-12q24.31 [35] and its molecular location has since been established as 12q24.23. Our original data [35], confirmed by various investigations [4, 5], indicate that H11/HspB8 is ubiquitously expressed in many tissues, being most abundant in human skeletal and smooth muscle, heart, and brain, with smaller expression in prostate, lung, kidney, and skin and virtually no signal detected in ovaries, testes, liver, pancreas, and spleen. While H11/HspB8 has two heat-shock-factor- (HSF-) binding sites, 1,000 bases upstream of the translation initiation site, its expression is not always heat inducible and it appears to differ in tumor as compared to normal cells [36, 37]. In melanocytes, where it is not heat inducible, H11/HspB8 causes cell-cycle arrest through the inhibition of cyclin E/Cdk2 and *β*-catenin phosphorylation at the transcriptional activity site Ser^552^ [38]. Consequently, H11/HspB8 is silenced in a large proportion of melanoma tissues by aberrant DNA methylation (a CpG island is present at the 5′ UTR, 216 bp upstream of the transcription start site) [36, 38]. H11/HspB8 is similarly silenced in prostate cancer and Ewing's sarcoma cells [36] and in hematologic malignancies [39]. H11/HspB8 is unique among all Hsps in terms of methylation-associated transcriptional repression in human cells. However, the mechanism by which H11/HspB8 acquires a new methylation pattern in some, but not other, cells and the relationship between altered methylation patterns and loss of accessibility to heat shock transcription factors are still unknown.

## 5. H11/HspB8 Is Membrane Associated and Has Intrinsic PK Activity

Confocal microscopy confirmed that H11/HspB8, like ICP10PK, is associated with the plasma membrane [35]. However, unlike ICP10PK, which has a TM and extracellular domain, H11/HspB8 forms tight complexes with phospholipids located in the intracellular leaflet of biological membranes [40]. H11/HspB8 has myristoylation motifs (Gly-Xaa-Xaa-Xaa-Ser/Thr; residues 62 and 132) that could enhance membrane-binding potential. However, it is unknown whether H11/HspB8 is myristoylated and the functional significance of its membrane-binding potential, whether myristoylation related or not, is unclear. Indeed, ICP10PK is myristoylated [41], but this modification does not seem to affect its membrane-binding potential which is TM-determined and is required for its signaling activity (Sections 2.1 and 3). However, H11/HspB8 retains catalytic motifs I–III and has intrinsic serine/threonine kinase activity, which favors Mn^2+^ ions, like ICP10PK [31, 36]. Kinase activity was lost upon mutation of the invariant Lys (Lys113) in catalytic motif II [31] but not Lys115 that is not located within a conserved catalytic motif. The Lys113 mutant retains all the other potential phosphorylation sites in H11/HspB8, supporting the interpretation that kinase activity is not a contamination artifact. Studies of the isolated H11/HspB8 protein confirmed that it undergoes autophosphorylation [42]. Like ICP10PK, H11/HspB8 has an SH3 module located between the PK catalytic motifs and a potential *N*-glycosylation site (amino acid 138), suggestive of potential structural similarity [14, 36]. Significantly, however, H11/HspB8 has cell-type-specific functions, which may or may not be kinase dependent (see Section 7).

## 6. H11/HspB8 and ICP10PK Inhibit UPR

Over 30% of cellular proteins are synthesized in the endoplasmic reticulum (ER). Delayed or impaired protein folding causes misfolded proteins accumulation thereby triggering ER stress that is characterized by distension and loss of homeostasis. To overcome the toxicity associated with the accumulation of misfolded proteins and/or their aggregation, the ER induces a cascade of reactions called the unfolded protein response (UPR). Cellular response to UPR restores normal cell function/survival and includes (i) translational attenuation, (ii) induction of ER-resident proteins that assist in protein folding, notably chaperones, (iii) induction of ER-associated degradation machinery, and (iv) ER enlargement to accommodate the large load of unfolded proteins. Translational attenuation is mediated by the double-stranded RNA-activated protein-kinase-like endoplasmic reticulum kinase (PERK) signaling pathway that reduces the activity of the ribosomal initiating factor (eIF2*α*) by phosphorylation of its *α*-subunit. Induction of ER-resident proteins is mediated by activated transcription factor 6 (ATF6) and inositol requiring kinase 1 (IRE1) receptors. ATF6 activates the transcription of molecular chaperones such as BiP, and IRE1 induces the synthesis of a potent X-box-binding protein 1 (XBP1) and consequently activates the transcription of ER-associated degradation proteins such as EDEM (reviewed in [43]). If the UPR response fails to restore cellular homeostasis, such as under conditions of prolonged UPR stress, the cell initiates apoptosis. Prolonged ER-stress-induced apoptosis is an important pathologic element of neurodegenerative diseases, diabetes, renal diseases, and atherosclerosis.

H11/HspB8 has basic chaperone activity. Original studies reported its interaction with HspB1 (HSP27) [33] but this proved to be assay dependent and was not seen in cross-linking and immunoprecipitation assays [31, 36] that in turn revealed its interaction with itself, HspB2, HspB6, *β*-crystallin, HspB3, and HspB7 [44]. Other proteins shown to interact with H11/HspB8 include Src-associated protein in mitosis 68 kDa (Sam68), which is involved in RNA transportation and processing [45], Ddx20, that has helicase activity [46], and *β*-crystallin mutants associated with the development of desmin-related cardiomyopathy or myofibrillar myopathy [47]. H11/HspB8 prevents in vivo aggregation of polyglutamine-containing proteins [48] and binds amyloid*β*-peptides (A 1–42 and A 1–40). However, it only inhibits the death of cardiovascular cells induced by Dutch-type A 1–40 mutants and the role that chaperone activity and/or UPR inhibition plays in protection is still poorly understood [49]. Two missense mutations, K141E and K141N, correlate with development of distal hereditary motor neuropathy type II (dHMN) [50] apparently related to decreased chaperone-like activity [48]. 

**Figure 2:**
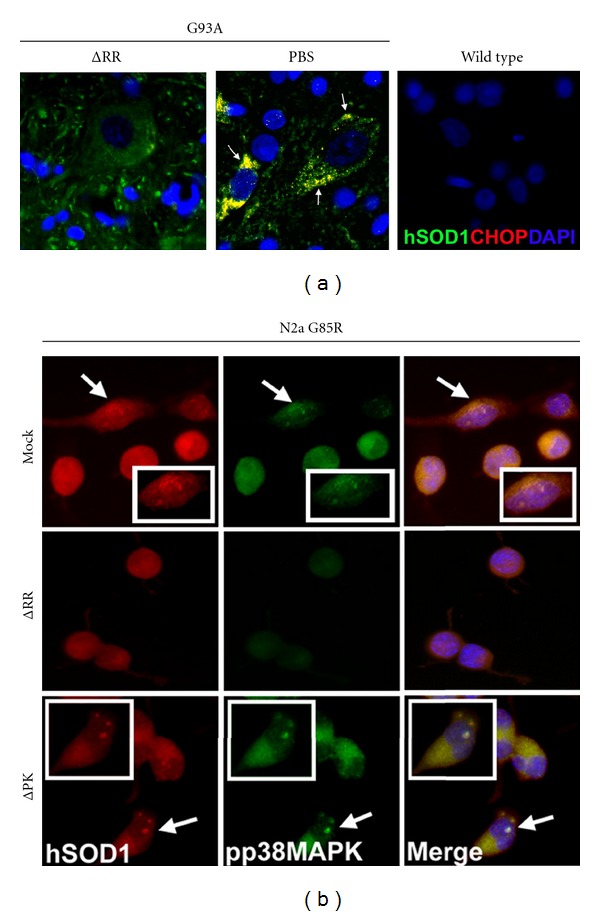
The ICP10PK vector ΔRR inhibits UPR. (a) mSOD1 (G93A) transgenic rats were treated with ΔRR (10^7^ pfu; 100 *μ*L) or an equal volume of PBS in both hind limbs by intramuscular injection beginning at age of 85 days. The ΔRR-treated G93A rats lived significantly longer than their PBS-treated counterparts with a median survival of 195 and 154 days, respectively (*P* < 0.001 by log-rank Mantel-Cox analysis). Serial sections from the L4-L5 region collected on day 153 were stained in double immunofluorescence with antibodies to human SOD1 (Alexa Fluor 488 conjugated secondary antibody; green) and CHOP (Alexa Fluor 594 conjugated secondary antibody; red). DAPI nuclear staining is blue. Only the PBS treated animals showed evidence of aggregates containing CHOP and mSOD1 co-localization. Scale bar = 10 *μ*m. (b). Neuronally differentiated N2a cells that were stably transfected with another mSOD1 mutant (G85R) were mock infected with PBS or infected with ΔRR or a vector that lacks ICP10PK (ΔPK) and treated with H_2_O_2_ (100 *μ*M; 2 hrs) to induce oxidative stress. N2a cells stably transfected with the wt SOD1 were studied in parallel and served as control. At 24 hrs after infection the cells were stained by double immunofluorescence with Alexa Fluor 594 conjugated SOD1 antibody (red) and Alexa Fluor 488 conjugated pp38MAPK antibody (green). DAPI nuclear stain is in blue. Scale bar = 10 *μ*m. Aggregates are not seen in the ΔRR-treated cells, associated with the inhibition of p38MAPK phosphorylation (activation).

The ability of H11/HspB8 to inhibit UPR is underscored by the finding that it can remove the misfolded mutant superoxide dismutase (mSOD1) [51] that is involved in the development of familial amyotrophic lateral sclerosis (ALS). ALS is a relatively common and fatal adult motor neuron (MN) disease with a prevalence of 2 per 100,000 individuals. mSOD1 misfolds within the MN endoplasmic reticulum (ER) causing protein aggregates that also contain the proapoptotic transcription factor C/EBP homologous protein (CHOP) and activated (phosphorylated) p38MAPK (pp38MAPK). These aggregates activate UPR, which disrupts the normal protein quality control function of the ER and plays a definitive role in disease initiation [52]. The mechanism whereby H11/HspB8 removes mSOD1 is still not fully understood and may involve interaction with the co-chaperone Bag-3, Hsc70, and CHIP (chaperone-associated ubiquitin ligase), thereby promoting autophagic removal by CHIP-mediated ubiquitylation [53]. However, its protective potential in mSOD1 transgenic animals is unclear. Interestingly, ICP10PK has strong protective activity in mSOD1 transgenic animals, as defined by significantly delayed disease onset and progression (*P* < 0.001 versus PBS), preserved neuromuscular junctions, and inhibition of MN degeneration (Laing et al., unpublished). Inhibition of mSOD1 aggregate formation (Figure 2(a)) is associated with protection, and studies of neuronally differentiated N2a cells that were stably transfected with the mSOD1 mutant G85R (N2aG85R) indicate that ICP10PK inhibits the formation of protein aggregates that contain mSOD1, pp38MAPK, and CHOP, apparently through its ability to inhibit p38MAPK phosphorylation (Figure 2(b)). The contribution of the *α*-crystallin domain, if any, is unclear, but H11/HspB8 and ICP10PK share the ability to inhibit UPR and regulate disease development. 

## 7. H11/HspB8 Is a Signaling Protein with Multiple Functions

Theoretical predictions and results of circular dichroism spectroscopy studies suggest that H11/HspB8 is an intrinsically disordered protein (IDP) [4]. IDPs are functional proteins that do not fold into stable tertiary structures under physiological conditions and are characterized by conformational flexibility, which allows them to quickly fluctuate among various conformational substates. This property allows the IDPs to respond reliably and swiftly to environmental changes and cellular signals. Intrinsic disorder is very high in signaling and cancer-related proteins [54]. While it is still unclear whether ICP10PK is an IDP and the contribution of intrinsic disorder to the H11/HspB8 signaling function is still unknown, H11/HspB8 displays a dual and opposing role in cell survival that is cell-type specific. In human skin, it is expressed in keratinocytes with long-term growth potential and its inhibition (with antisense oligonucleotides) inhibited DNA synthesis and cell proliferation in cultured human keratinocytes [32]. H11/HspB8 has antiapoptotic activity in glioblastoma and breast cancer cells through cell-cycle regulation, potentially involving activation of the growth-associated transcription factor E2F and/or the cyclin-dependent kinase cdk4. In glioblastoma cells, H11/HspB8 expression is inversely correlated to Sam68, thereby increasing the S phase population and the expression of cell-cycle regulatory proteins such as cyclins E and A, ribonucleotide reductase, and proliferating cell nuclear antigen (PCNA), which are required for the transition from the G1 to the S phase of the cell-cycle [55]. In transgenic mice with increased cardiac expression, H11/HspB8 imparts myocardial hypertrophy accompanied by activation of Akt and p70S6 kinase [56, 57]. In these animals, H11/HspB8 directly interacts with bone morphogenetic protein (BMP) and activates both its “canonical” and “noncanonical” signaling pathways leading to regulation of activity and intracellular location of different PKs (including Akt, AMP-dependent protein kinase, and the *ε*-isoform of PKC) and inducible nitric oxide synthase [56–58]. However, its effect is dose dependent such that low doses (delivered with an adenovirus vector) increased the cell size, while high doses induced apoptosis of cardiac myocytes [59]. In these experiments, the hypertrophic effect was attributed to the activation of PI3-K and Akt and was independent of the H11/HspB8 kinase activity. By contrast, the proapoptotic effect was attributed to the inhibition of casein kinase 2 and it depended on the H11/HspB8 kinase activity [59]. The contribution of intrinsic disorder to these different functions is still unclear. 

## 8. H11/HspB8 Is a Tumor Suppressor

In human melanocytes, H11/HspB8 causes growth arrest through *β*-catenin phosphorylation at the transcriptional activity site Ser^552^ and the resulting inhibition of the cell-cycle regulatory proteins cyclin E/Cdk2 [38]. Accordingly, H11/HspB8 expression is silenced through DNA methylation in a high proportion (77%) of melanoma tissues and cultures [38]. Significantly, H11/HspB8 is also silenced in a high proportion of atypical nevi, but not in most benign nevi, suggesting that its silencing contributes to tumor progression [60]. H11/HspB8 is also silenced by aberrant DNA methylation in prostate cancer and Ewing's sarcoma cells [36] and in hematologic malignancies [39] and its restored expression (e.g., by treatment with the demethylating agent Aza-C) induced cell death in all these cancers and inhibited tumor growth in xenograft models [36–39]. In melanoma, there is a good correlation between the levels of H11/HspB8 methylation, restored expression, and Aza-C-induced cell death, which is apparently related to the ability of high levels of restored H11/HspB8 to overcome the cell survival functions of the constitutively activated PI3-K/Akt pathway. Indeed, PI3-K inhibition increased Aza-C-induced cell death in the melanoma lines that had low/moderate levels of H11/HspB8 methylation and thereby restored expression [38]. While the effectiveness of Aza-C treatment in patients with metastatic melanoma is controversial, it is generally believed that future testing of Aza-C, in combination with other agents, is warranted [61]. However, the identification of molecular and prognostic markers remains a crucial aspect of patient selection for individual-targeted treatments. H11/HspB8 appears to be such a marker for Aza-C treatment of melanoma, with patients whose tumors have high levels of H11/HspB8 DNA methylation being seriously considered for demethylation therapy [38]. 

**Figure 3:**
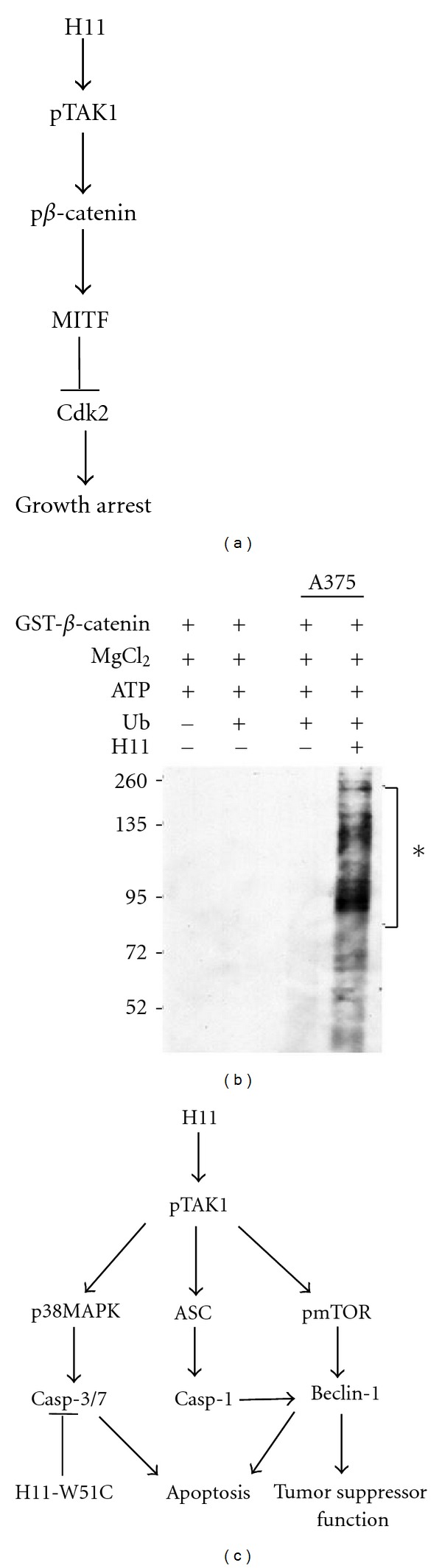
H11/HsPB8 causes growth arrest and melanoma cell death through distinct TAK1-regulated pathways. (a) Schematic representation of H11/HspB8-induced growth arrest involves *β*-catenin phosphorylation by the activated TAK1. (b) GST-*β*-catenin phosphorylated when mixed with cell extracts containing H11/HspB8-activated TAK1 is ubiquitylated causing its degradation (∗). Ubiquitylation is not seen when GST-*β*-catenin is mixed with cell extracts that lack H11/HspB8 and therefore pTAK1. (c) H11/HspB8 induces different death pathways in the genetically diverse melanoma cells A375 and A2058 that initiate with TAK1 activation. In A375 cells, the TAK1/p38MAPK pathway activates caspase-3/7 to cause apoptosis. In A2058 cells, the TAK1/p38MAPK pathway activates caspase-3, but TAK1 also activates caspase-1 through ASC upregulation and upregulates Beclin-1 through mTOR phosphorylation at S2481 (pmTORS2481). Caspase-1 cleaves Beclin-1 to promote apoptosis, but Beclin-1 also contributes to cell death through still unknown tumor suppressor functions. The W51C mutant of H11/HspB8 has dominant-negative activity. It inhibits the death-inducing potential of the wild-type H11/HspB8 by inhibiting caspase activation through the activation of the B-Raf/MEK/ERK pathway [33].

The cell-cycle regulatory potential of H11/HspB8 in normal cells, its dysfunctional state in cancer cells, and its ability to induce tumor cell death upon restored expression identify H11/HspB8 as a tumor suppressor. Also characteristic of tumor suppressors is the cell-type specificity of the H11/HspB8 effects and the finding that it can undergo single-site transforming mutation. Indeed, one of the H11/HspB8 missense mutants, W51C, causes anchorage-independent growth and it overrides the death-inducing capacity of the wild-type H11/HspB8 (Figure 3(c)) through activation of the B-Raf/MEK/ERK pathway [36], which is a notorious contributor to melanoma development. The frequency of H1/HpB8 mutation is still unknown, but, given our finding that most studied melanoma cultures are sensitive to Aza-C-mediated cell killing, it is likely that such mutations are relatively rare. 

## 9. Restored H11/HspB8 Expression Induces Tumor Cell Death through TAK1 Activation

Restored expression of H11/HspB8 causes growth arrest and inhibits tumor growth through the activation of programmed cell death pathways. It overrides the genetic diversity of melanoma tumors, a recognized obstacle to the development of effective therapeutic strategies, through the activation of distinct death pathways. However, all the inhibitory pathways initiate with the binding and direct phosphorylation (activation) of transforming growth factor *β*-activated kinase 1 (TAK1). TAK1 is not activated by the missense H11/HspB8 mutants P173H and W51C, which do not cause cell death. Growth arrest is through the ability of activated TAK1 (pTAK1) to bind and phosphorylate *β*-catenin (Figure 3(a)) promoting its ubiquitylation and proteosomal degradation (Figure 3(b)). The outcome is reduced nuclear accumulation and inhibition of transcriptional activity, as evidenced by decreased levels of the transcription factor MITF and its target CDK2 that functions in the G1/S and G2/M transitions (Figure 3(a)). This results in growth arrest in G1 and particularly G2 [37]. 

The death pathways initiated by pTAK1 include p38MAPK/caspases-3/7-dependent apoptosis, which is a major component of cell death in one of the studied melanoma cultures/xenografts (A375) and previously unrecognized death pathways in the other studied melanoma culture/xenografts (A2058) [37, 62]. In A2058 cells, the activated TAK1 caused upregulation of the inflammasome component apoptosis-associated speck-like protein containing a CARD (ASC) and it activated caspase-1 without producing IL-1*β* and IL-18, indicating that caspase-1 functions independent of the inflammasome-mediated death pathway (pyroptosis) [63]. Indeed, caspase-1 cleaved the haploinsufficient tumor suppressor Beclin-1 that was also upregulated by H11/HspB8 through a TAK1/mTORC1 pathway that involved mTOR phosphorylation at S2481, which is the site of intrinsic mTORC1-specific catalytic activity [64]. The cleaved Beclin-1 fragment translocated to the mitochondria and contributed to apoptosis, but the pronounced upregulation of Beclin-1 in A2058 xenografts, which have lower levels of H11/HspB8-induced apoptosis than their A375 counterparts, suggests that Beclin-1 also contributes to tumor growth inhibition through still unrecognized tumor suppressor functions (Figure 3(c)). These could include proliferative senescence, regulation of autophagic cell death networks, and cell-type-specific nonapoptotic cell death induced by deregulated Ras activation. Collectively the data identify TAK1 as a central player in the H11/HspB8-induced growth arrest/death of melanoma cells and underscore the therapeutic promise of H11/HspB8. It is still unclear whether the same pathways are used by the restored H11/HspB8 to cause cell death in prostate cancer, Ewing sarcoma, and/or hematologic malignancies.

## 10. H11/HspB8, Inflammation, and Autoimmune Disorders

**Figure 4:**
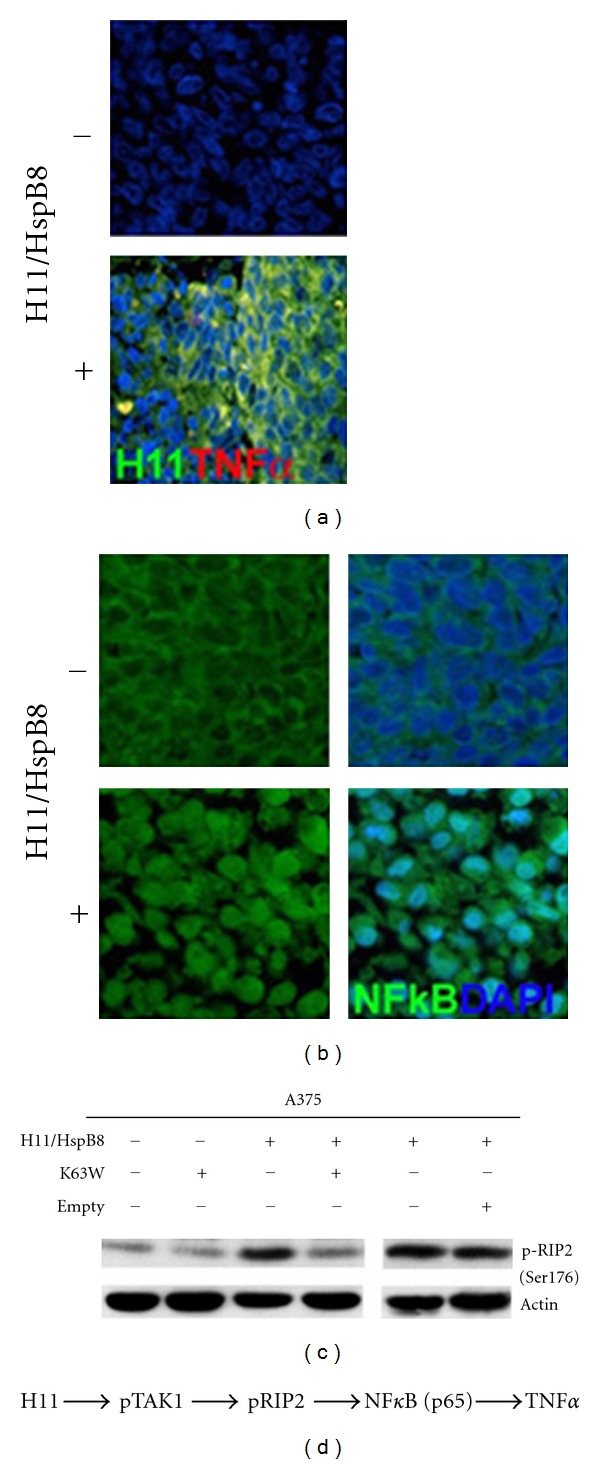
H11/HspB8 induces TAK1-dependent TNF-*α* expression. (a) Serial sections from A2058 and A375 xenografts collected at the end of the follow-up period from mice subjected or not to restored H11/HspB8 expression were stained with antibodies to H11/HspB8 (Alexa Fluor 488 conjugated secondary antibody; green) and TNF-*α* (Alexa Fluor 594 conjugated secondary antibody; red). (b). Serial sections from the A2058 and A375 xenografts as in (a) were stained with Alexa Fluor 488 conjugated NF-*κ*B p65 antibody. Restored H1/HspB8 activates NF-*κ*B (intranuclear localization). (c) A375 cells stably transfected with tet-inducible H11/HspB8 were given Dox to restore H11/HspB8 expression alone or with the TAK1 dominant-negative mutant K63W or empty vector. Extracts collected 3 days later were immunoblotted with antibody to pRIP2 (Ser176). They were stripped and reprobed with antibody to actin (loading control). (d) Schematic representation of the pathways involved in H11/HspB8-induced TNF-*α* induction.

H11/HspB8 was implicated in inflammatory processes based on its ability to activate dendritic cells through a Toll-like receptor-4- (TLR4-) dependent pathway [65]. Its restored expression in melanoma cells induces the proinflammatory cytokine TNF-*α* (Figure 4(a)), which likely contributes to tumor growth inhibition [66]. This pathway also initiates with H11/HspB8-mediated TAK1 activation. It includes pTAK1-mediated phosphorylation of receptor-interacting protein 2 kinase (RIP-2) (inhibited by the dominant-negative TAK1 vector K63W (Figure 4(c))) that activates NF-*κ*B [67] causing its intranuclear localization (Figure 4(b)). NF-*κ*B induces TNF-*α* by binding kB promoter elements [68] (Figure 4(d)). The data confirm that TAK1 activation is the pivotal factor in the ability of H11/HspB8 to regulate cell life/death decisions including inflammatory pathways other than pyroptosis (see also Section 8).

The ability of H11/HspB8 to induce cytokine production is consistent with findings for ICP10PK, except that the latter decreases the levels of TNF-*α* while increasing expression of the anti-inflammatory cytokine IL-10, at least in microglia [27]. Notwithstanding their apparently different inflammation modulatory functions and the contribution of the cell type, the similarity between ICP10PK and H11/HspB8 suggests that they may contribute to the onset of autoimmunity through “molecular mimicry.” Indeed, linear peptide epitopes, processed from viral proteins, mimic normal host self-proteins, thus leading to an immune cross-reaction between the virus and host cell antigens, with inflammation as a typical sign of autoimmune diseases [69, 70]. In addition, because chaperones can broadly associate with other proteins including autoantigens and recruit antigen presentation pathways, they can work as endogenous adjuvants in immune responses, for example, those previously induced by viral infection. However, additional studies are needed in order to better understand the possible interaction between H11/HspB8 and ICP10PK in inflammation and autoimmunity. 

## 11. H11/HspB8 and the Origin of ICP10PK

H11/HspB8 and ICP10PK are atypical PKs that lack some of the catalytic motifs of the eukaryotic PKs [9, 10] and share 32% identity and 59% homology [14, 31]. They share multifunctional activity that encompasses signaling, UPR, and the regulation of life-cycle potential. However, their exact relationship remains unclear. The level of sequence homology between H11/HspB8 and ICP10PK is similar to that seen for viral Bcl-2 homologues and their cellular counterparts, supporting the interpretation that the two proteins are members of the same protein family. However, if we accept the premise that the presence of an *α*-crystallin motif, even if degenerate, is a *sine qua non *criterion for evolutionary-based inclusion into the sHsp family [1], we must infer that ICP10PK is evolutionarily related to H11/HspB8. According to this interpretation, ICP10PK is likely to have evolved from H11/HspB8, which was originally captured by HSV-2 in order to provide functional advantages favorable to virus survival, such as the inhibition of neuronal apoptosis. The finding that ICP10PK is required for HSV-2 growth and latency reactivation [16–19] supports this interpretation. Presumably, once it was captured, H11/HspB8 fell under the control of the viral R1 promoter, losing the regulatory constraints that define its cell-type-specific death-inducing potential while retaining kinase and ATPase-independent chaperone activity and the ability to inhibit UPR. This interpretation is supported by the finding that a missense mutation (namely, W51C) can convert H11/HspB8 from a proapoptotic to a dominant antiapoptotic protein that induces cell proliferation through the B-Raf pathway independent of the cell type. It is consistent with current understanding of virus evolution, which recognizes viruses as “gene robbers” that have evolved after cellular species [71]. However, the presence of a TM domain and its contribution to the ICP10PK kinase activity are distinct from the processes used by H11/HspB8, and the possibility cannot be excluded that H11/HspB8 evolved from ICP10PK captured by the cell from HSV-2 for a specific function. Indeed, recent studies have shown that cells contain genes of viral origin such as the syncitins, which are of retroviral origin and function in placentation. In simians, *syncytin-1* and *-2* entered the primate genome 25 and >40 Mya, respectively, and retained their coding capacity in all of the subsequent lineages. They display placenta-specific expression, are fusogenic, and may have immunosuppressive activity. A pair of env genes from endogenous retroviruses that have a completely distinct origin are also essential for placentation in the mouse, suggesting that, on several occasions in the course of mammalian evolution, retroviral infections have resulted in the independent capture of genes that have been positively selected for a convergent physiological role [72]. However, HSV DNA does not integrate, presumably reducing the likelihood of gene capture. Notwithstanding, the finding that HSV-2 is the only virus that contains Hsp homologues suggests that it has likely evolved differently than its closely related homologue, HSV-1.

## 12. Conclusion and Perspectives

The involvement of H11/HspB8 in the regulation of multiple cellular processes is unquestionable, but, despite the finding that it inhibits UPR and stimulates signaling, the molecular mechanism(s) of its multivariate activities remain largely unknown. Its similarity to ICP10PK, which is expressed by a common viral pathogen, underscores the evolutionary importance of these functions, particularly as it pertains to human disorders and therapeutic strategies. Future investigation should include the potential interaction of H11/HspB8 with ATP-dependent chaperones and their co-chaperones, as well as different components of proteasomal and autophagosomal systems. Further analysis of the signaling pathways modulated by H11/HspB8 should emphasize the contribution of the cell type, the criteria that define new methylation patterns in some tumors, and the genetic diversity of distinct tumor lines. Better understanding of the ability of H11/HspB8 to modulate regulatory protein families (namely Bcl-2 and IAP), its mutation frequency, and its contribution to disease should also be emphasized. The mechanism of the H11/HspB8-induced death of tumor cells other than melanoma (namely prostate) and the role of TAK1 need further investigation, as does the contribution of H11/HspB8 to oncolytic virotherapy. Because H11/HspB8 is involved in different cellular processes, it appears to be an attractive therapeutic target. The use of H11/HspB8 as a marker for the selection of patients to be included in Aza-C treatment protocols should be further explored, particularly within the context of panels of hypermethylated genes and combinatorial therapy that includes PI3-K inhibition. The identification of hypermethylated genes that may contribute to H11/HspB8-induced cell death is particularly important. Finally, better understanding of the inflammatory potential of H11/HspB8 and the contribution that molecular mimicry with ICP10PK may have towards the development of autoimmune disorders should also be given further attention. Such understanding will provide important new tools to advance the development of much needed therapeutic strategies.
